# 2-[1-(4-Bromo­phen­yl)-3-hy­droxy-3-(4-meth­oxy­phen­yl)prop­yl]cyclo­hexa­nol

**DOI:** 10.1107/S1600536813015869

**Published:** 2013-06-12

**Authors:** Ísmail Çelik, Mehmet Akkurt, Hayreddin Gezegen, Muhammed M. Üremiş, Narcis Duteanu

**Affiliations:** a Department of Physics, Faculty of Sciences, Cumhuriyet University, 58140 Sivas, Turkey; b Department of Physics, Faculty of Sciences, Erciyes University, 38039 Kayseri, Turkey; c Department of Physics, Faculty of Arts and Sciences, Gaziosmanpaşa University, 60240 Tokat, Turkey; d Faculty of Industrial Chemistry and, Environmental Engineering, Politehnica University of Timisoara, 6 Pirvan Boulevard, 300223, Timimisoara, Romania

## Abstract

In the title compound, C_22_H_27_BrO_3_, the cyclo­hexane ring adopts a chair conformation. The dihedral angle between the benzene rings is 41.9 (4)°. In the crystal, mol­ecules are linked by O—H⋯O and C—H⋯O hydrogen bonds, forming a three-dimensional network. In addition, π–π stacking inter­actions [centroid–centroid distance = 3.953 (6) Å] between the benzene rings of the meth­oxy­benzene groups occur.

## Related literature

For the biological properties of 1,5-diols, see: Flamme & Roush (2005 ▶); Hansen *et al.* (2003 ▶); Huang *et al.* (2009 ▶); Oger *et al.* (2010 ▶). For details of the synthesis, see: Ceylan & Gezegen (2008 ▶); Gezegen *et al.* (2010 ▶). For ring conformation analysis, see: Cremer & Pople (1975 ▶). For standard bond-length data, see: Allen *et al.* (1987 ▶).
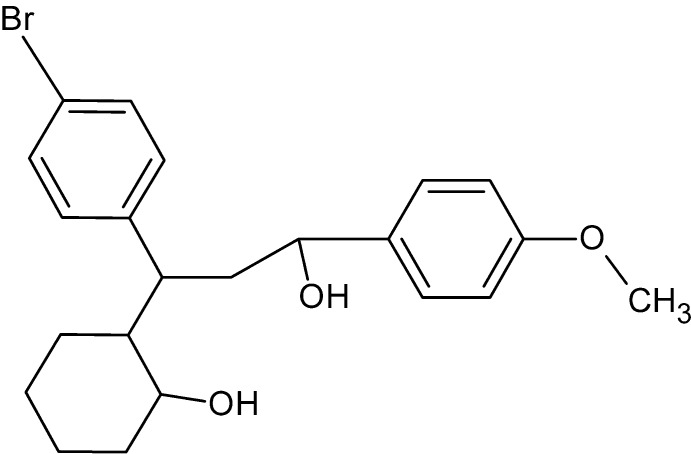


## Experimental

### 

#### Crystal data

C_22_H_27_BrO_3_
*M* *_r_* = 419.34
Monoclinic, 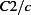

*a* = 23.2993 (14) Å
*b* = 10.9282 (5) Å
*c* = 22.3632 (11) Å
β = 133.032 (3)°
*V* = 4162.2 (4) Å^3^
*Z* = 8
Mo *K*α radiation
μ = 1.99 mm^−1^
*T* = 296 K
0.60 × 0.34 × 0.28 mm

#### Data collection

Stoe IPDS 2 diffractometer
Absorption correction: integration [*X-RED32* (Stoe & Cie, 2002 ▶) and *XABS2* (Parkin *et al.*, 1995 ▶)] *T*_min_ = 0.448, *T*_max_ = 0.572
4301 measured reflections
4301 independent reflections
2327 reflections with *I* > 2σ(*I*)
*R*_int_ = 0.000

#### Refinement

*R*[*F* ^2^ > 2σ(*F* ^2^)] = 0.068
*wR*(*F* ^2^) = 0.198
*S* = 1.03
4301 reflections
240 parameters
149 restraints
H atoms treated by a mixture of independent and constrained refinement
Δρ_max_ = 0.78 e Å^−3^
Δρ_min_ = −0.56 e Å^−3^

### 

Data collection: *X-AREA* (Stoe & Cie, 2002 ▶); cell refinement: *X-AREA*; data reduction: *X-RED32* (Stoe & Cie, 2002 ▶); program(s) used to solve structure: *SHELXS97* (Sheldrick, 2008 ▶); program(s) used to refine structure: *SHELXL97* (Sheldrick, 2008 ▶); molecular graphics: *ORTEP-3 for Windows* (Farrugia, 2012 ▶); software used to prepare material for publication: *WinGX* (Farrugia, 2012 ▶) and *PLATON* (Spek, 2009 ▶).

## Supplementary Material

Crystal structure: contains datablock(s) global, I. DOI: 10.1107/S1600536813015869/sj5331sup1.cif

Structure factors: contains datablock(s) I. DOI: 10.1107/S1600536813015869/sj5331Isup2.hkl

Supplementary material file. DOI: 10.1107/S1600536813015869/sj5331Isup3.cml

Additional supplementary materials:  crystallographic information; 3D view; checkCIF report

## Figures and Tables

**Table 1:** Hydrogen-bond geometry (Å, °)

*D*—H⋯*A*	*D*—H	H⋯*A*	*D*⋯*A*	*D*—H⋯*A*
O1—H1*A*⋯O2^i^	0.82	2.13	2.854 (4)	147
O2—H2*A*⋯O2^ii^	0.78 (3)	2.46 (2)	2.871 (6)	115 (2)
C5—H5⋯O3^iii^	0.93	2.36	3.287 (10)	171

Symmetry codes: (i) 
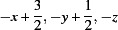
; (ii) 
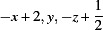
; (iii) 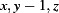.

**Fig. 1.**
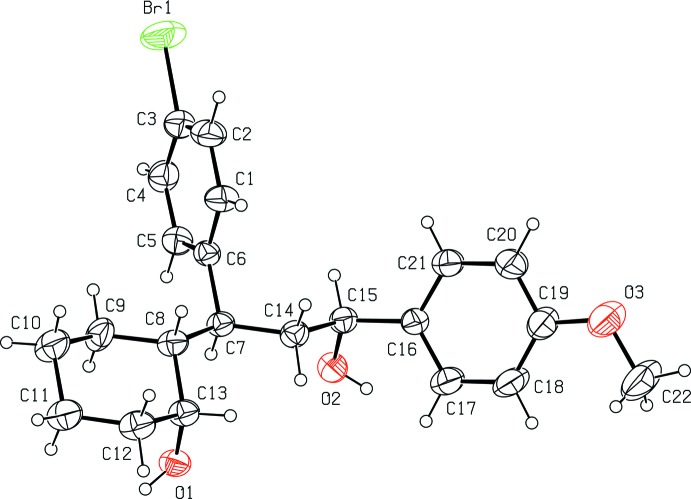
The title molecule with the atom numbering scheme. Displacement ellipsoids for non-H atoms are drawn at the 30% probability level.

**Fig. 2.**
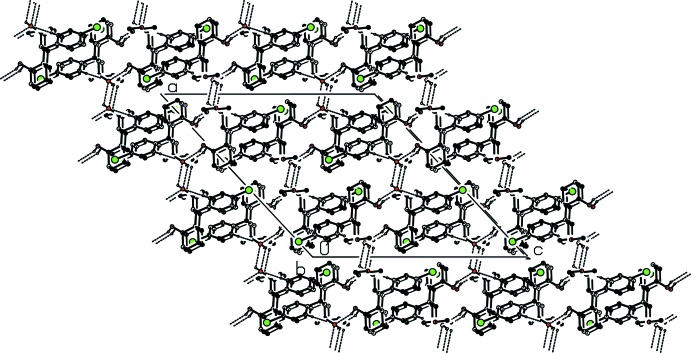
The packing and hydrogen bonding of the title compound viewed down the b axis. H atoms not involved in hydrogen bonding are omitted for clarity. Hydrogen bonds are shown as dashed lines.

## supplementary crystallographic information

### Comment

1,5-Diols are useful and important compounds as intermediates in synthetic organic reactions (Huang *et al.*, 2009; Oger *et al.*, 2010). They are used in the synthesis of bioactive molecules and natural products (Flamme & Roush, 2005; Hansen *et al.*, 2003). In this paper we report synthesis of 2-(1-(4-bromophenyl)-3-hydroxy-3-(4-methoxyphenyl)propyl)cyclohexanol in moderate yield together with its crystal structure.

The cyclohexyl ring of the title compound (I, Fig. 1) adopts a chair conformation with the puckering parameters (Cremer & Pople, 1975) of Q_T_ = 0.564 (6) Å, θ = 2.7 (6) ° and φ = 12 (13)°. The two benzene rings (C1–C6 and C16–C21) make a dihedral angle of 41.9 (4)° with each other. The C6–C7–C8–C13, C6–C7–C14–C15, C7–C14–C15–O2 and C7–C14–C15–C16 torsion angles are 177.9 (4), 59.2 (6), 64.0 (5) and -171.5 (4)°, respectively. All bond lengths of (I) are within normal ranges (Allen *et al.*, 1987), as are the bond angles.

In the crystal, intermolecular O—H···O and C—H···O hydrogen bonds connect molecules, forming a three-dimensional network (Table 1, Fig. 2). Furthermore, π-π stacking interactions [*Cg*3···*Cg*3(2 - *x*, *y*, 1/2 - *z*) = 3.953 (6) Å, where *Cg*3 is a centroid of the C16–C21 benzene ring], between the benzene rings of the methoxybenzene groups, stabilize the crystal packing.

### Experimental

2-(1-(4-Bromophenyl)-3-hydroxy-3-(4-methoxyphenyl)propyl)cyclohexanol was synthesized from 2-(1-(4-bromophenyl)-3-(4-methoxyphenyl)-3-oxopropyl)cyclohexanone (1,5-diketone) (Ceylan & Gezegen, 2008; Gezegen *et al.*, 2010). To a solution of 2-(1-(4-bromophenyl)-3-(4-methoxyphenyl)-3-oxopropyl)cyclohexanone (1 mmol) in THF-MeOH (12 ml 5:1) was added NaBH_4_ (3 mmol) and stirred for 16 h at room temperature. After completion of the reaction, the mixture was transferred to a separatory funnel, dilute HCl (10% 10 ml) was added and extracted with diethyl ether (3x20 ml). The organic layer was dried over Na_2_SO_4_ and evaporated. The crude product was crystallized from EtO_2_-hexane (3:1) to give pure 2-(1-(4-bromophenyl)-3-hydroxy-3-(4-methoxyphenyl)propyl)cyclohexanol in 75% yield; m.p.: 470–472 K.

### Refinement

C-bound H atoms and the hydroxyl H atom H1A were positioned geometrically and refined using a riding model with O—H = 0.82 Å, C—H = 0.93 Å (aromatic), 0.96 Å (methyl), 0.97 Å (methylene) and 0.98 Å (methine), and with *U*_iso_(H) = 1.5*U*_eq_(C_methyl_, O_hydroxyl_), and *U*_iso_(H) = 1.2*U*_eq_(C_aromatic, methylene, methine_). The other hydroxyl H atom H2A was found in difference Fourier maps and the O2—H2A bond length were restrained to 0.83 (2) Å with *U*_iso_(H) = 1.5*U*_eq_(O). Twelve poorly fitted reflections (2 2 1), (0 2 2), (-25 3 19), (-9 3 7), (-10 2 20), (-13 7 5), (-6 2 6), (2 0 0), (-6 2 10), (10 6 2), (-9 11 3) and (-15 5 6) were omitted from the refinement owing to bad disagreement between Fo and Fc.

### Crystal data

**Table:**

C_22_H_27_BrO_3_	*F*(000) = 1744
*M**_r_* = 419.34	*D*_x_ = 1.338 Mg m^−^^3^
Monoclinic, *C*2/*c*	Mo *K*α radiation, λ = 0.71073 Å
Hall symbol: -C 2yc	Cell parameters from 538 reflections
*a* = 23.2993 (14) Å	θ = 1.8–28.0°
*b* = 10.9282 (5) Å	µ = 1.99 mm^−^^1^
*c* = 22.3632 (11) Å	*T* = 296 K
β = 133.032 (3)°	Prism, colourless
*V* = 4162.2 (4) Å^3^	0.60 × 0.34 × 0.28 mm
*Z* = 8	

### Data collection

**Table:**

Stoe IPDS 2 diffractometer	4301 independent reflections
Radiation source: sealed X-ray tube, 12 x 0.4 mm long-fine focus	2327 reflections with *I* > 2σ(*I*)
Plane graphite monochromator	*R*_int_ = 0.000
Detector resolution: 6.67 pixels mm^-1^	θ_max_ = 26.5°, θ_min_ = 2.0°
ω scans	*h* = −29→21
Absorption correction: integration [*X-RED32* (Stoe & Cie, 2002) and *XABS2* (Parkin *et al.*, 1995)]	*k* = 0→13
*T*_min_ = 0.448, *T*_max_ = 0.572	*l* = 0→28
4301 measured reflections	

### Refinement

**Table:**

Refinement on *F*^2^	Primary atom site location: structure-invariant direct methods
Least-squares matrix: full	Secondary atom site location: difference Fourier map
*R*[*F*^2^ > 2σ(*F*^2^)] = 0.068	Hydrogen site location: inferred from neighbouring sites
*wR*(*F*^2^) = 0.198	H atoms treated by a mixture of independent and constrained refinement
*S* = 1.03	*w* = 1/[σ^2^(*F*_o_^2^) + (0.0945*P*)^2^ + 0.8672*P*] where *P* = (*F*_o_^2^ + 2*F*_c_^2^)/3
4301 reflections	(Δ/σ)_max_ = 0.001
240 parameters	Δρ_max_ = 0.78 e Å^−^^3^
149 restraints	Δρ_min_ = −0.56 e Å^−^^3^

### Special details

**Table:**

Geometry. Bond distances, angles *etc*. have been calculated using the rounded fractional coordinates. All su's are estimated from the variances of the (full) variance-covariance matrix. The cell e.s.d.'s are taken into account in the estimation of distances, angles and torsion angles
Refinement. Refinement on *F*^2^ for ALL reflections except those flagged by the user for potential systematic errors. Weighted *R*-factors *wR* and all goodnesses of fit *S* are based on *F*^2^, conventional *R*-factors *R* are based on *F*, with *F* set to zero for negative *F*^2^. The observed criterion of *F*^2^ > σ(*F*^2^) is used only for calculating -*R*-factor-obs *etc*. and is not relevant to the choice of reflections for refinement. *R*-factors based on *F*^2^ are statistically about twice as large as those based on *F*, and *R*-factors based on ALL data will be even larger.

### Fractional atomic coordinates and isotropic or equivalent isotropic displacement parameters (Å^2^)

**Table:**

	*x*	*y*	*z*	*U*_iso_*/*U*_eq_	
Br1	0.90669 (5)	0.12894 (8)	0.49681 (4)	0.1178 (4)	
O1	0.68319 (18)	0.2009 (3)	−0.02218 (19)	0.0645 (10)	
O2	0.91572 (17)	0.3614 (3)	0.19089 (19)	0.0594 (10)	
O3	0.9041 (4)	0.9462 (3)	0.2060 (3)	0.130 (3)	
C1	0.7850 (3)	0.2963 (4)	0.2729 (3)	0.0658 (16)	
C2	0.8135 (3)	0.2666 (5)	0.3495 (3)	0.0762 (19)	
C3	0.8642 (3)	0.1688 (5)	0.3903 (3)	0.0678 (16)	
C4	0.8854 (3)	0.1024 (4)	0.3576 (3)	0.0664 (16)	
C5	0.8566 (3)	0.1340 (4)	0.2812 (3)	0.0579 (16)	
C6	0.8059 (2)	0.2320 (4)	0.2374 (2)	0.0479 (11)	
C7	0.7740 (2)	0.2639 (4)	0.1536 (2)	0.0494 (12)	
C8	0.6854 (2)	0.2325 (4)	0.0871 (2)	0.0548 (14)	
C9	0.6696 (3)	0.0990 (5)	0.0910 (3)	0.0761 (16)	
C10	0.5823 (3)	0.0706 (6)	0.0323 (3)	0.090 (2)	
C11	0.5419 (3)	0.1067 (5)	−0.0550 (3)	0.0787 (18)	
C12	0.5591 (3)	0.2377 (5)	−0.0597 (3)	0.0705 (16)	
C13	0.6465 (2)	0.2654 (4)	0.0002 (2)	0.0565 (14)	
C14	0.7910 (2)	0.3971 (4)	0.1491 (2)	0.0542 (12)	
C15	0.8779 (2)	0.4304 (4)	0.2098 (2)	0.0541 (12)	
C16	0.8874 (3)	0.5678 (4)	0.2089 (3)	0.0542 (12)	
C17	0.8778 (4)	0.6232 (5)	0.1470 (4)	0.092 (3)	
C18	0.8820 (5)	0.7492 (5)	0.1436 (4)	0.108 (3)	
C19	0.8971 (4)	0.8204 (4)	0.2031 (4)	0.087 (3)	
C20	0.9063 (4)	0.7675 (4)	0.2643 (4)	0.088 (2)	
C21	0.9017 (3)	0.6423 (4)	0.2674 (3)	0.0690 (19)	
C22	0.8947 (6)	1.0051 (5)	0.1428 (6)	0.146 (5)	
H1	0.75060	0.36190	0.24470	0.0790*	
H1A	0.65010	0.16080	−0.06370	0.0970*	
H2	0.79880	0.31140	0.37260	0.0910*	
H2A	0.9441 (7)	0.398 (3)	0.1907 (8)	0.0890*	
H4	0.91900	0.03590	0.38580	0.0800*	
H5	0.87170	0.08820	0.25880	0.0700*	
H7	0.80120	0.21210	0.14380	0.0600*	
H8	0.65930	0.28140	0.09940	0.0660*	
H9A	0.69390	0.07980	0.14630	0.0910*	
H9B	0.69360	0.04740	0.07780	0.0910*	
H10A	0.55890	0.11540	0.04870	0.1080*	
H10B	0.57480	−0.01610	0.03430	0.1080*	
H11A	0.55980	0.05310	−0.07400	0.0950*	
H11B	0.48570	0.09600	−0.09090	0.0950*	
H12A	0.53430	0.29160	−0.04850	0.0850*	
H12B	0.53620	0.25450	−0.11480	0.0850*	
H13	0.65320	0.35340	−0.00170	0.0670*	
H14A	0.76880	0.41340	0.09420	0.0650*	
H14B	0.76450	0.44980	0.15880	0.0650*	
H15	0.90090	0.40710	0.26470	0.0650*	
H17	0.86820	0.57490	0.10680	0.1100*	
H18	0.87460	0.78490	0.10100	0.1280*	
H20	0.91570	0.81630	0.30430	0.1050*	
H21	0.90830	0.60750	0.30980	0.0830*	
H22A	0.93450	0.97650	0.14400	0.2190*	
H22B	0.89960	1.09200	0.15120	0.2190*	
H22C	0.84380	0.98630	0.09060	0.2190*	

### Atomic displacement parameters (Å^2^)

**Table:**

	*U*^11^	*U*^22^	*U*^33^	*U*^12^	*U*^13^	*U*^23^
Br1	0.1390 (7)	0.1527 (7)	0.0648 (4)	−0.0010 (5)	0.0707 (5)	0.0173 (4)
O1	0.0527 (17)	0.092 (2)	0.0556 (17)	−0.0169 (16)	0.0396 (15)	−0.0152 (16)
O2	0.0511 (17)	0.0657 (18)	0.0653 (18)	−0.0043 (14)	0.0412 (15)	−0.0047 (14)
O3	0.260 (6)	0.060 (2)	0.194 (5)	0.006 (3)	0.204 (5)	0.002 (3)
C1	0.075 (3)	0.075 (3)	0.060 (2)	0.008 (2)	0.051 (3)	0.002 (2)
C2	0.095 (4)	0.084 (3)	0.071 (3)	0.001 (3)	0.065 (3)	−0.008 (3)
C3	0.071 (3)	0.076 (3)	0.053 (2)	−0.011 (2)	0.041 (2)	−0.003 (2)
C4	0.062 (3)	0.066 (3)	0.057 (2)	0.003 (2)	0.035 (2)	0.010 (2)
C5	0.055 (3)	0.063 (3)	0.056 (2)	0.002 (2)	0.038 (2)	0.000 (2)
C6	0.042 (2)	0.056 (2)	0.0412 (19)	−0.0040 (17)	0.0266 (18)	−0.0032 (17)
C7	0.046 (2)	0.060 (2)	0.045 (2)	−0.0022 (18)	0.0321 (19)	−0.0002 (18)
C8	0.040 (2)	0.078 (3)	0.046 (2)	−0.003 (2)	0.0292 (18)	0.000 (2)
C9	0.061 (3)	0.091 (3)	0.056 (2)	−0.026 (3)	0.032 (2)	0.006 (2)
C10	0.061 (3)	0.139 (5)	0.057 (3)	−0.037 (3)	0.035 (3)	−0.002 (3)
C11	0.048 (3)	0.125 (4)	0.055 (2)	−0.022 (3)	0.032 (2)	−0.005 (3)
C12	0.046 (2)	0.106 (4)	0.050 (2)	0.001 (2)	0.029 (2)	0.005 (2)
C13	0.045 (2)	0.075 (3)	0.046 (2)	−0.0012 (19)	0.0297 (19)	0.0007 (19)
C14	0.053 (2)	0.058 (2)	0.048 (2)	0.0022 (19)	0.033 (2)	0.0056 (18)
C15	0.053 (2)	0.062 (2)	0.047 (2)	−0.005 (2)	0.034 (2)	−0.0005 (19)
C16	0.057 (2)	0.059 (2)	0.053 (2)	−0.006 (2)	0.040 (2)	−0.0039 (19)
C17	0.159 (6)	0.068 (3)	0.101 (4)	−0.007 (3)	0.109 (4)	−0.002 (3)
C18	0.209 (7)	0.065 (3)	0.135 (5)	0.006 (4)	0.151 (6)	0.013 (3)
C19	0.147 (6)	0.053 (3)	0.127 (5)	0.002 (3)	0.119 (5)	−0.001 (3)
C20	0.140 (5)	0.063 (3)	0.099 (4)	−0.005 (3)	0.097 (4)	−0.011 (3)
C21	0.093 (4)	0.064 (3)	0.068 (3)	0.001 (2)	0.062 (3)	0.000 (2)
C22	0.287 (12)	0.068 (4)	0.227 (9)	0.018 (5)	0.232 (10)	0.028 (5)

### Geometric parameters (Å, º)

**Table:**

Br1—C3	1.904 (5)	C19—C20	1.361 (11)
O1—C13	1.436 (7)	C20—C21	1.378 (6)
O2—C15	1.426 (7)	C1—H1	0.9300
O3—C19	1.381 (6)	C2—H2	0.9300
O3—C22	1.427 (13)	C4—H4	0.9300
O1—H1A	0.8200	C5—H5	0.9300
O2—H2A	0.78 (3)	C7—H7	0.9800
C1—C2	1.389 (8)	C8—H8	0.9800
C1—C6	1.374 (9)	C9—H9A	0.9700
C2—C3	1.378 (8)	C9—H9B	0.9700
C3—C4	1.341 (10)	C10—H10A	0.9700
C4—C5	1.387 (8)	C10—H10B	0.9700
C5—C6	1.387 (7)	C11—H11A	0.9700
C6—C7	1.512 (5)	C11—H11B	0.9700
C7—C8	1.550 (6)	C12—H12A	0.9700
C7—C14	1.530 (6)	C12—H12B	0.9700
C8—C9	1.522 (7)	C13—H13	0.9800
C8—C13	1.524 (5)	C14—H14A	0.9700
C9—C10	1.521 (10)	C14—H14B	0.9700
C10—C11	1.531 (7)	C15—H15	0.9800
C11—C12	1.510 (8)	C17—H17	0.9300
C12—C13	1.520 (8)	C18—H18	0.9300
C14—C15	1.524 (7)	C20—H20	0.9300
C15—C16	1.520 (6)	C21—H21	0.9300
C16—C21	1.375 (8)	C22—H22A	0.9600
C16—C17	1.382 (10)	C22—H22B	0.9600
C17—C18	1.386 (8)	C22—H22C	0.9600
C18—C19	1.364 (10)		
			
C19—O3—C22	118.0 (6)	C14—C7—H7	107.00
C13—O1—H1A	110.00	C7—C8—H8	107.00
C15—O2—H2A	116 (2)	C9—C8—H8	107.00
C2—C1—C6	122.2 (5)	C13—C8—H8	107.00
C1—C2—C3	118.1 (6)	C8—C9—H9A	109.00
Br1—C3—C2	119.0 (5)	C8—C9—H9B	109.00
Br1—C3—C4	119.2 (4)	C10—C9—H9A	109.00
C2—C3—C4	121.8 (5)	C10—C9—H9B	109.00
C3—C4—C5	119.2 (5)	H9A—C9—H9B	108.00
C4—C5—C6	121.7 (6)	C9—C10—H10A	110.00
C5—C6—C7	121.0 (5)	C9—C10—H10B	110.00
C1—C6—C5	117.0 (4)	C11—C10—H10A	110.00
C1—C6—C7	122.0 (4)	C11—C10—H10B	110.00
C6—C7—C14	111.8 (3)	H10A—C10—H10B	108.00
C6—C7—C8	110.2 (4)	C10—C11—H11A	109.00
C8—C7—C14	112.5 (3)	C10—C11—H11B	109.00
C7—C8—C13	114.5 (4)	C12—C11—H11A	109.00
C7—C8—C9	111.9 (4)	C12—C11—H11B	109.00
C9—C8—C13	110.0 (3)	H11A—C11—H11B	108.00
C8—C9—C10	112.3 (5)	C11—C12—H12A	109.00
C9—C10—C11	110.1 (6)	C11—C12—H12B	109.00
C10—C11—C12	111.8 (4)	C13—C12—H12A	109.00
C11—C12—C13	112.8 (5)	C13—C12—H12B	109.00
O1—C13—C12	110.7 (4)	H12A—C12—H12B	108.00
O1—C13—C8	110.6 (4)	O1—C13—H13	108.00
C8—C13—C12	110.5 (5)	C8—C13—H13	108.00
C7—C14—C15	114.5 (3)	C12—C13—H13	108.00
O2—C15—C16	113.2 (5)	C7—C14—H14A	109.00
C14—C15—C16	109.9 (4)	C7—C14—H14B	108.00
O2—C15—C14	108.9 (3)	C15—C14—H14A	109.00
C17—C16—C21	117.5 (5)	C15—C14—H14B	109.00
C15—C16—C17	121.4 (5)	H14A—C14—H14B	108.00
C15—C16—C21	121.0 (5)	O2—C15—H15	108.00
C16—C17—C18	121.4 (7)	C14—C15—H15	108.00
C17—C18—C19	119.6 (8)	C16—C15—H15	108.00
O3—C19—C20	116.3 (6)	C16—C17—H17	119.00
O3—C19—C18	123.9 (7)	C18—C17—H17	119.00
C18—C19—C20	119.8 (5)	C17—C18—H18	120.00
C19—C20—C21	120.5 (6)	C19—C18—H18	120.00
C16—C21—C20	121.1 (6)	C19—C20—H20	120.00
C2—C1—H1	119.00	C21—C20—H20	120.00
C6—C1—H1	119.00	C16—C21—H21	119.00
C1—C2—H2	121.00	C20—C21—H21	119.00
C3—C2—H2	121.00	O3—C22—H22A	109.00
C3—C4—H4	120.00	O3—C22—H22B	110.00
C5—C4—H4	120.00	O3—C22—H22C	110.00
C4—C5—H5	119.00	H22A—C22—H22B	109.00
C6—C5—H5	119.00	H22A—C22—H22C	109.00
C6—C7—H7	107.00	H22B—C22—H22C	109.00
C8—C7—H7	107.00		
			
C22—O3—C19—C20	−179.9 (10)	C9—C8—C13—C12	56.1 (6)
C22—O3—C19—C18	0.2 (16)	C7—C8—C13—O1	60.3 (5)
C2—C1—C6—C5	0.7 (9)	C7—C8—C13—C12	−176.9 (4)
C2—C1—C6—C7	179.3 (6)	C8—C9—C10—C11	55.8 (7)
C6—C1—C2—C3	−0.2 (10)	C9—C10—C11—C12	−53.1 (8)
C1—C2—C3—Br1	178.1 (5)	C10—C11—C12—C13	53.9 (8)
C1—C2—C3—C4	−0.7 (10)	C11—C12—C13—C8	−55.2 (6)
Br1—C3—C4—C5	−177.8 (5)	C11—C12—C13—O1	67.6 (6)
C2—C3—C4—C5	1.1 (10)	C7—C14—C15—C16	−171.5 (4)
C3—C4—C5—C6	−0.5 (10)	C7—C14—C15—O2	64.0 (5)
C4—C5—C6—C7	−179.0 (5)	O2—C15—C16—C17	44.5 (8)
C4—C5—C6—C1	−0.4 (9)	C14—C15—C16—C21	98.9 (7)
C5—C6—C7—C8	109.2 (6)	O2—C15—C16—C21	−139.1 (6)
C1—C6—C7—C8	−69.4 (6)	C14—C15—C16—C17	−77.5 (9)
C1—C6—C7—C14	56.5 (7)	C15—C16—C21—C20	−176.6 (7)
C5—C6—C7—C14	−125.0 (5)	C17—C16—C21—C20	0.0 (12)
C14—C7—C8—C9	178.6 (4)	C15—C16—C17—C18	176.3 (8)
C14—C7—C8—C13	52.5 (5)	C21—C16—C17—C18	−0.3 (13)
C6—C7—C14—C15	59.2 (6)	C16—C17—C18—C19	1.0 (16)
C8—C7—C14—C15	−176.3 (4)	C17—C18—C19—C20	−1.4 (16)
C6—C7—C8—C9	−56.0 (5)	C17—C18—C19—O3	178.5 (10)
C6—C7—C8—C13	177.9 (4)	O3—C19—C20—C21	−178.9 (9)
C13—C8—C9—C10	−57.8 (7)	C18—C19—C20—C21	1.0 (15)
C7—C8—C9—C10	173.7 (5)	C19—C20—C21—C16	−0.3 (14)
C9—C8—C13—O1	−66.8 (6)		

### Hydrogen-bond geometry (Å, º)

**Table:**

*D*—H···*A*	*D*—H	H···*A*	*D*···*A*	*D*—H···*A*
O1—H1*A*···O2^i^	0.82	2.13	2.854 (4)	147
O2—H2*A*···O2^ii^	0.78 (3)	2.46 (2)	2.871 (6)	115 (2)
C5—H5···O3^iii^	0.93	2.36	3.287 (10)	171

Symmetry codes: (i) −*x*+3/2, −*y*+1/2, −*z*; (ii) −*x*+2, *y*, −*z*+1/2; (iii) *x*, *y*−1, *z*.
